# Kinematic changes in 5-m swimming start performance using the new double kick start block

**DOI:** 10.3389/fspor.2025.1720289

**Published:** 2026-01-06

**Authors:** Ivan Matúš, Tomáš Eliaš, Bibiana Vadašová, Wojcieh Czarny, Łukasz Rydzik, Tadeusz Ambroży, Kacper Szczypka, Pavel Ružbarský

**Affiliations:** 1 Faculty of Sport, University of Presov, Presov, Slovakia; 2 Department of Sport Theory and Motor Skills, Institute of Sports Sciences, University of Physical Culture in Krakow, Krakow, Poland; 3 Independent Researcher, Krakow, Poland

**Keywords:** kick start, pedal, performance swimmers, starting platform OSB12, swimming

## Abstract

Enhancing start performance can substantially reduce overall race time, particularly in sprint swimming events. The aim of this study was to examine differences in angular and temporal characteristics of individual start phases over 5 m when performing from the new DKS (“double kick start”) starting block in various pedal angle settings and from the OSB12 starting platform. The sample consisted of 13 junior competitive swimmers (age 17.7 ± 1.1 years), who completed a total of 10 maximal-effort starts. From the DKS block, swimmers performed two attempts at pedal settings of 30°/30°, 40°/40°, 50°/50°, and 60°/60°, and two attempts from the OSB12 platform. Kinematic parameters above and below the water surface were analyzed using a 4-camera SwimPro system and Dartfish ProSuite 4.0 software. Differences between conditions were evaluated using repeated measures ANOVA. For significant interactions, *post hoc* LSD analysis was applied, and effect sizes were determined by partial eta squared. Results showed that the DKS block with 40°/40° and 50°/50° pedal settings enabled junior performance swimmers to execute significantly more effective starts compared to the OSB12 platform (*p* < .001, *η*p² = 0.78–0.90). Improvements were most evident in shorter block time (0.62 ± 0.04–0.06 s vs. 0.78 ± 0.01 s), enhanced flight phase parameters (shorter flight time and greater flight distance; *p* < .001), and faster 5-m times (1.44 ± 0.06–0.08 s vs. 1.70 ± 0.03 s; *p* < .001). Our findings suggest that the use of variable pedal angles on the new DKS starting block significantly improves start kinematics and positively affects 5-m performance, which may contribute to optimizing swimmers’ overall performance in the initial phase of the race.

## 1 Introduction

In sprint disciplines in athletics and swimming, the start phase represents a key moment that significantly influences overall performance, particularly acceleration and subsequent race progression (1–3). Biomechanical studies have systematically investigated the factors affecting start performance, with the aim of optimizing technique and enhancing athlete efficiency. In particular, anthropometric characteristics and starting-block settings have a significant impact on start performance. Biomechanical analysis helps refine athletic technique by providing detailed insights into the mechanics of human movement, such as optimizing starting posture and acceleration gait in sprinting (4, 5) In athletics, Cavedon et al. (6, 7) demonstrated that adjusting block settings according to individual anthropometry leads to improved start efficiency. Bezodis et al. (8) found that effective coordination and distribution of force between the front and rear lower limbs in the set position contribute to faster block clearance. In another study, Bezodis et al. (9) emphasized the importance of lower limb joint biomechanics, particularly at the ankle and knee, for force and velocity generation. Milanese et al. (10) highlighted that varying rear knee angles markedly influence start kinematics, supporting the need for individually tailored techniques. Comprehensive kinetic and kinematic start models in athletics were presented by Čoh et al. (11), and a systematic review by Valamatos et al. (12) further underlined the complexity of start optimization. These findings confirm that a detailed understanding of lower limb movements and positions in the set stance is crucial for optimizing the start in athletics.

In swimming, start performance is equally critical, especially in 50 m events. Takeda et al. (13) reported that the vertical force impulse generated by the front lower limb during the kick start was significantly higher than that of the rear leg, while the horizontal impulse was significantly greater for the rear leg compared to the front. The horizontal impulses from the arms and the rear leg also contributed to faster block clearance and shorter block time, thereby improving take-off velocity compared with grab starts (14). Similar advantages of the kick start over the grab start were demonstrated by Taladriz et al. (15), who observed statistically significant differences in all temporal variables except flight time, as well as greater vertical take-off velocity in the kick start. Although no significant differences were found in the angular momentum of the center of mass at take-off, shorter block time and more effective rotational involvement of the lower limbs during the block and flight phases of the kick start were crucial for improved 5-m performance. Matúš et al. (16) examined foot placement and its influence on the set position, emphasizing that correct foot placement, particularly a narrower stance on the starting block, significantly enhances start stability and efficiency. Suito et al. (17) found that adopting the preferred lower-limb position on the block resulted in significantly shorter block time, higher horizontal take-off velocity, and faster 10-m performance compared with switching limb positions. Rudnik et al. (18) highlighted gender differences in start kinematics, with men achieving higher horizontal take-off velocity, longer flight distance, and shorter block time than women. Performance in the kick start over 5 m, 10 m, and 15 m in men was strongly correlated with block time, horizontal take-off velocity, and flight distance. In the study by Shepherd et al. (19), it was shown that although 5-m time differed between sexes, body position at take-off was similar and did not affect performance. The start performance was mainly influenced by the horizontal velocity of the center of mass and the trunk angle at take-off. Studies by Achurra et al. (20, 21) demonstrated that optimizing the kick plate position based on tibia length improves block movement and enhances push-off efficiency. Barlow et al. (22) found that neutral- and rear-weighted kick start positions resulted in faster 15-m times compared to front-weighted starts. However, the starting position had no effect on swimmer velocity in the 4.5–5.5 m and 14.5–15.5 m segments. Beretić et al. (23) confirmed that the use of a kick plate leads to significant changes in start kinematics among elite swimmers, translating into faster 10-m performance. Honda et al. (24) reported that kick plate position had no effect on 7.5-m time. Rear-weighted starts resulted in higher horizontal take-off velocity, but no significant differences were observed in 7.5-m times. Studies by Kibele et al. (25, 26) showed that modifying the start stance of elite swimmers on the OSB11 block—particularly front-weighted positioning, narrower stance, and higher center of mass—can reduce start time by an average of 0.06 s and improve overall performance by up to 0.14 s. Sakai et al. (27) examined the effect of center of mass positioning on torque generation in all four limbs during the start, demonstrating that appropriate weight distribution enhances total force and push-off velocity. Durović et al. (28) concluded that swimmers with superior underwater kicking possess greater potential for faster 10-m start performance. Tor et al. (29) compared three underwater trajectories after the start and found that the most effective one occurred at a maximum dive depth of −0.91 ± 0.12 m, at which swimmers reached the shortest 15-m time. All of these findings confirm that a detailed understanding of lower-limb movements, positions, and kinematic-dynamic moments is crucial for optimizing the swimming start. The aim of our study was to examine and understand the differences in angular and temporal characteristics of individual start phases over 5 m when performing from the new DKS (“double kick start”) block at various pedal angle settings, and to compare them with those from the OSB12 starting platform. Performance during the block phase can influence technique and efficiency in subsequent phases of the start and maximize swimming performance in sprint events. The new DKS starting block was designed based on sprinting techniques in athletics, where athletes achieve shorter block times.

## 2 Materials and methods

### 2.1 Participants

The research sample consisted of 13 male junior competitive swimmers who regularly participated in the highest-level national competitions in Slovakia and trained at least five times per week for 2–3 h per day. The swimmers’ age, height, body mass, and sport age were 17.7 ± 1.1 years, 184.4 ± 6.2 cm, 79.2 ± 3.3 kg, and 11 ± 2 years, respectively. The mean performance level in the 50-m freestyle was 595 ± 45 World Aquatics points. Only male swimmers were included in the study to ensure a homogeneous sample, as recent research has reported clear sex-related differences in swimming start kinematics and performance determinants (18, 19). All participants were healthy, without lower limb problems or injuries at the time of testing. Swimmers were informed about the purpose and procedures of the study and provided written informed consent prior to participation. Ethical approval for this study was obtained from the Ethics Committee of the University of Presov, Presov, Slovakia (Approval No. ECUP042022PO).

### 2.2 Testing procedures

The testing of start jumps from the OSB12 starting platform and the new double kick start (DKS) block was conducted at the University of Presov swimming pool (6-lane facility) during the main competitive season, three weeks prior to the Slovak National Championships. A medical doctor from the Faculty of Sports, University of Presov, placed markers on the left and right sides of each swimmer's body at the following anatomical points: lateral margin of the transverse tarsal joint, lateral malleolus, lateral knee condyle, greater trochanter, lateral margin of the scapular spine, elbow epicondyle, ulnar styloid process, and medial side of the 5th metacarpal–phalangeal joint.

A standardized warm-up followed according to the RAMP protocol—raise, activate, mobilize, and potentiate (30)—after which the swimmers completed a 400 m swim under coach supervision. Following the warm-up, each swimmer performed two practice starts in their preferred position. The starting procedure followed World Aquatics rules (31): all starts were performed under the supervision of the same experimenter, who consistently gave the standard “Take your marks” command and initiated the start using an auditory signal. From the OSB12 platform, swimmers started from their individually preferred basic position in random order. This stance corresponded to each swimmer's usual kick start, had been previously optimised in training with respect to stance width, foot height, kick plate level and centre-of-mass position, and was the position they routinely used in competition. This condition therefore served as the reference competition start for the comparison with the DKS block configurations. In contrast, on the DKS block the pedal positions were standardised according to leg length: the front pedal was positioned at 45% of the distance from the greater trochanter to the lateral malleolus measured from the front edge, and the rear pedal at 60% of this distance (6, 32). Both pedals on the DKS block were set at the same angle. Each swimmer performed a total of 10 maximal-effort 5-m starts: two from the OSB12 platform and two from each of the four pedal angle settings on the DKS block (30°/30°, 40°/40°, 50°/50°, and 60°/60°; front/rear pedal). The interval between attempts was 45 s, with a rest period of 9.45 min between two series of starts. A 30-min break was provided between OSB12 and DKS testing conditions. According to Tor et al. (29), swimmers should glide underwater to a distance of 6.6 m; therefore, they were instructed not to perform undulatory movements or kicks underwater, since the measured distance was limited to 5 m. The load during a single start lasted approximately 0.5–0.7 s (block phase), indicating that the recovery period between attempts was sufficient.

### 2.3 Data collection

#### 2.3.1 Anthropometric data

Anthropometric data were collected by a medical doctor from the Faculty of Sports, University of Presov. Body mass was measured to the nearest 0.1 kg using a certified electronic scale (Salter 9150, UK). Body height was measured to the nearest 0.1 cm using a portable stadiometer (Seca 213, Hamburg, Germany).

#### 2.3.2 Kinematic data

Kinematic parameters were recorded using four cameras integrated into the SwimPro system (SwimPro IQ Lab v250319, Australia). The cameras captured the start signal, the swimmer's set position, the push-off phase, the flight phase, and the underwater phase at 5 m. The first two cameras were placed opposite each other, 1.6 m from the OSB12 starting platform or the DKS block, at a height of 1.5 m. These cameras recorded the set position on the block. The third camera was positioned 1.6 m from the pool edge at a height of 1.5 m and captured the above-water phase. The fourth camera was located 5 m from the pool edge at a depth of −1.7 m to capture the underwater phase. According to Bartlett (33), in order to minimize potential technological errors in the 2D kinematic analysis (e.g., parallax errors caused by visual distortion), the optical axis of each camera was aligned perpendicularly to the swimmer's plane of movement. In addition, each camera was centered along an imaginary line perpendicular to the ground, passing through the hip joint of the leg facing the camera in the “take your marks” position. Cameras 1–3 (above-water phase) were mounted on tripods, whereas the fourth camera (underwater phase) was mounted on a telescopic pole. For enhanced visibility of the markers, additional halogen and LED lighting was used. The SwimPro system recorded video at 50 Hz with a shutter speed of 1/1,000 s.

#### 2.3.3 Instrumentation

**Figure 1:**
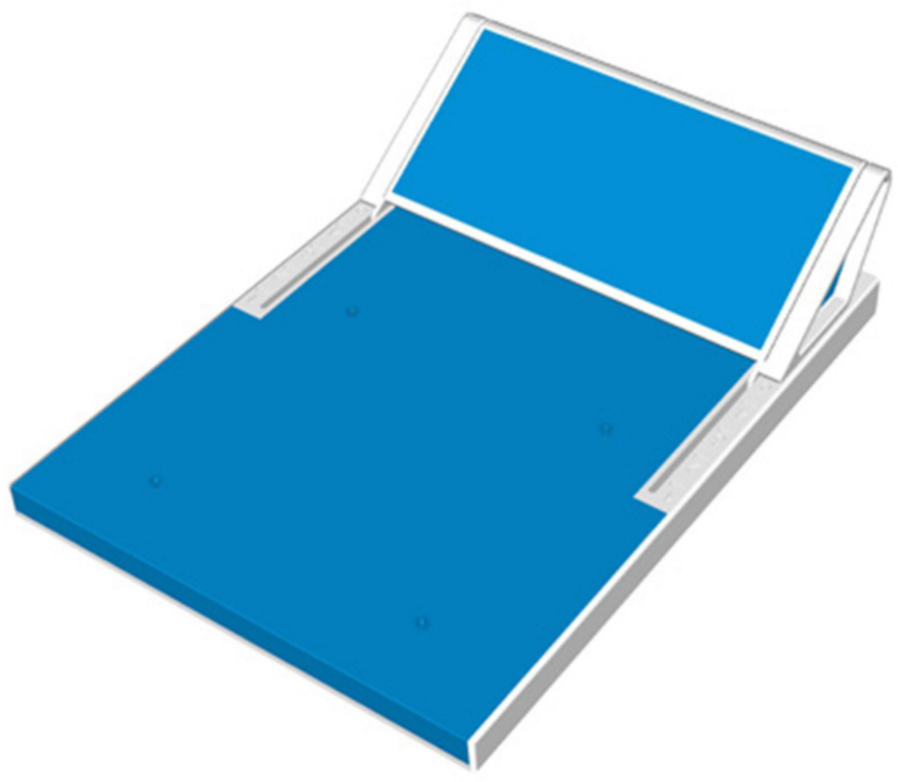
Starting platform OSB12.

For testing, the OSB12 starting platform (345.507.2 Version 1.4, March 2020, Swiss Timing Ltd.) with a 5° inclination was used. Its dimensions were 740 × 520 × 38 mm. The kick plate was adjustable in the anterior–posterior direction in five positions, within a range of 200 mm, and set at an angle of 30° (Figure 1).

**Figure 2:**
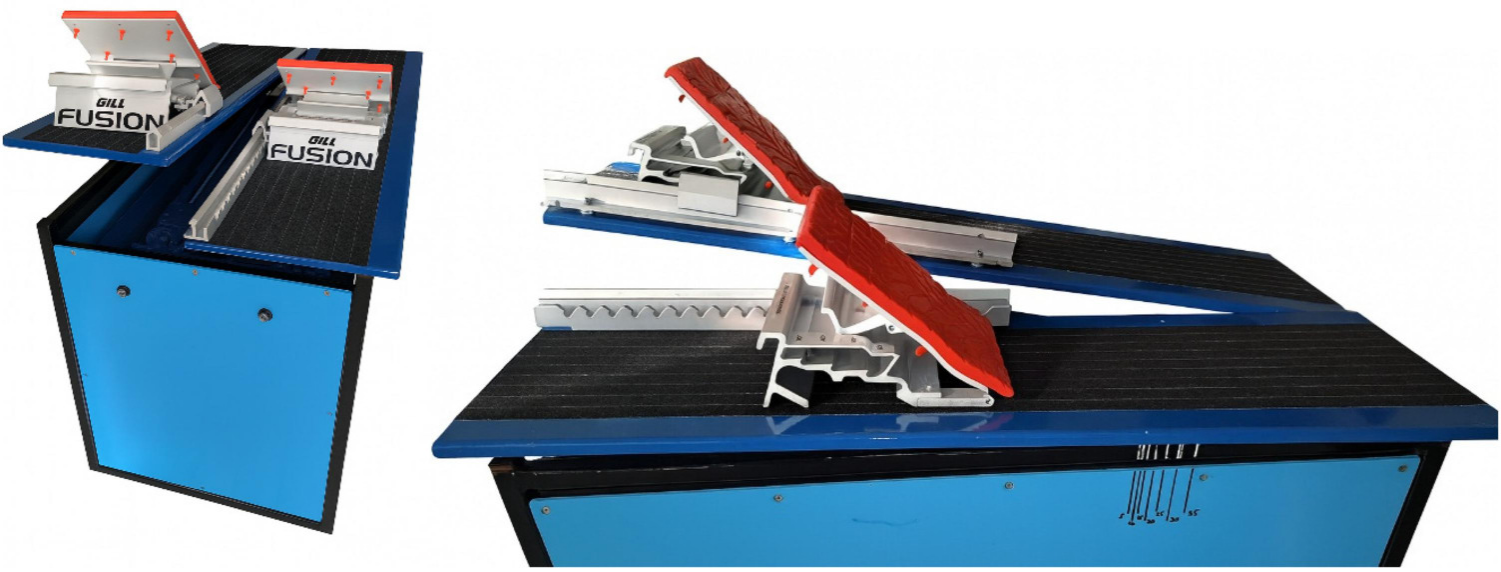
New DKS starting block—double kick start.

The new DKS starting block (patent SK 288741) had dimensions of 1100 × 620 × 700 mm (Figure 2). It contained two independent foot pedals adjustable within a range of 0–25°. Gill Fusion F10 starting blocks were mounted on the pedals, with integrated markers to enable precise foot placement. The pedals measured 200 × 265 mm and were adjustable within an angular range of 30°–60°. Angle spikes at the base of the pedals ensured a non-slip surface. The pedals could be moved in the anterior–posterior direction over a range of 510 mm with increments of 30 mm. The height of the front edge of the platform above the water surface was the same as for the OSB12—70 cm, with an inclination of 10°. The DKS device used in this study has not yet received World Aquatics approval; consequently, the conclusions apply only to training scenarios.

#### 2.3.4 Data analysis

Kinematic outputs were stored on a laptop connected to the SwimPro cameras and subsequently exported for further analysis using Dartfish software (Dartfish ProSuite 4.0, 2005; Fribourg, Switzerland). A single experienced analyst specializing in kinematic analysis manually digitized the markers and quantified angular, linear, and temporal characteristics at specific video frames in each trial. First, frames corresponding to the set position during the “take your marks” command on both the OSB12 platform and the DKS block were evaluated to directly measure sagittal plane angles using the digital goniometer tool in Dartfish. Videos from the first and second cameras were used to estimate the following joint and trunk angles: front knee angle (FKA,°), front ankle angle (FAA,°), rear knee angle (RKA,°), rear ankle angle (RAA,°), front hip angle (FHA,°), and rear hip angle (RHA,°). Next, the video was paused at the frame corresponding to the swimmer's last foot contact with the OSB12 platform or DKS block. These frames, captured by the third camera, were used to estimate temporal and angular variables: block time (BT, s) and take-off angle (TA,°°). From the same camera, flight phase parameters were analyzed: entry angle (EA,°), flight time (FT, s), and flight distance (FD, m). The length of the starting block was used as a reference for calibrating flight distance. The fourth camera was used to evaluate underwater phase variables: glide time (GT, s), glide distance (GD, m), and time to 5 m (T5, s). Angles were measured with an accuracy of 0.1°, and time was measured with an accuracy of 0.01 s.

#### 2.3.5 Statistical analysis

Normality of the data was assessed using the Shapiro–Wilk test. Descriptive statistics (mean ± SD) were calculated for all variables. Differences between conditions were examined using repeated measures ANOVA. When a significant main effect was detected, *post hoc* analysis (LSD) was conducted. Effect size was determined using partial eta squared (*η*p²) and interpreted according to Cohen (34) as small (0.01), medium (0.06), and large (0.14). Statistical analyses were performed in SPSS (version 27.0.1.0, IBM Corp., Armonk, NY, USA). The level of statistical significance was set at *p* < .05.

## 3 Results

The mean (SD) values of the kinematic parameters measured during the 5-m start from the DKS starting block and the OSB12 platform are presented in Table 1. Five starting conditions were analysed: DKS30°/30°, DKS40°/40°, DKS50°/50°, DKS60°/60° and OSB12. For all variables, repeated-measures ANOVA indicated a significant main effect of starting condition (*p* < .01).

**Table 1:** Mean (SD) values of kinematic variables across DKS starting block configurations and the OSB12 platform.

Phases	Variables	Starting block/platform					RM ANOVA	
		DKS30°/30°	DKS40°/40°	DKS50°/50°	DKS60°/60°	OSB12		
		Mean (SD)	Mean (SD)	Mean (SD)	Mean (SD)	Mean (SD)	F	*p*
Block	FKA (°)	104.4 (2.1)^2^^,^^3^^,^^4^^,^^5^	100.7 (3.1)^1^^,^^3^^,^^4^^,^^5^	96.3 (2.9)^1^^,^^2^^,^^4^^,^^5^	94.1 (3.6)^1^^,^^2^^,^^3^^,^^5^	133.0 (2.2)^1^^,^^2^^,^^3^^,^^4^	1,083.10	<.01
	FAA (°)	97.2 (2.0)^2^^,^^3^^,^^4^^,^^5^	99.7 (2.9)^1^^,^^3^^,^^4^^,^^5^	101.5 (3.2)^1^^,^^2^^,^^4^^,^^5^	103.4 (3.2)^1^^,^^2^^,^^3^^,^^5^	129.7 (2.5)^1^^,^^2^^,^^3^^,^^4^	582.40	<.01
	FHA (°)	51.6 (2.3)^3^^,^^4^^,^^5^	50.2 (2.2)^3^^,^^4^^,^^5^	48.6 (1.8)^1^^,^^2^^,^^4^^,^^5^	46.4 (2.0)^1^^,^^2^^,^^3^^,^^5^	39.6 (2.7)^1^^,^^2^^,^^3^^,^^4^	70.70	<.01
	RKA (°)	125.4 (2.2)^2^^,^^3^^,^^4^^,^^5^	123.0 (3.2)^1^^,^^3^^,^^4^^,^^5^	120.8 (3.1)^1^^,^^2^^,^^5^	119.8 (3.3)^1^^,^^2^^,^^5^	84.2 (3.4)^1^^,^^2^^,^^3^^,^^4^	1,339.50	<.01
	RAA (°)	92.0 (1.7)^2^^,^^3^^,^^4^^,^^5^	95.2 (2.1)^1^^,^^3^^,^^4^^,^^5^	101.4 (3.3)^1^^,^^2^^,^^4^^,^^5^	104.0 (3.1)^1^^,^^2^^,^^3^^,^^5^	86.5 (2.3)^1^^,^^2^^,^^3^^,^^4^	178.30	<.01
	RHA (°)	81.5 (1.8)^3^^,^^4^^,^^5^	80.4 (1.9)^4^^,^^5^	79.6 (1.9)^1^^,^^4^^,^^5^	77.6 (2.6)^1^^,^^2^^,^^3^^,^^5^	52.5 (2.7)^1^^,^^2^^,^^3^^,^^4^	512.90	<.01
	BT (s)	0.68 (0.02)^2^^,^^3^^,^^5^	0.62 (0.04)^1^^,^^4^^,^^5^	0.62 (0.06)^1^^,^^4^^,^^5^	0.66 (0.08)^2^^,^^3^^,^^5^	0.78 (0.01)^1^^,^^2^^,^^3^^,^^4^	22.54	<.01
Flight	TA (°)	33.4 (2.5)^3^^,^^4^^,^^5^	32.2 (1.3)^5^	32.1 (1.4)^1^^,^^5^	31.4 (1.9)^1^^,^^5^	35.4 (1.0)^1^^,^^2^^,^^3^^,^^4^	10.54	<.01
	EA (°)	36.9 (1.3)^2^^,^^3^^,^^4^	35.7 (1.2)^1^^,^^3^^,^^4^^,^^5^	34.5 (1.8)^1^^,^^2^^,^^4^^,^^5^	33.0 (1.5)^1^^,^^2^^,^^3^^,^^5^	37.6 (0.7)^2^^,^^3^^,^^4^	24.20	<.01
	FT (s)	0.33 (0.02)^2^^,^^3^^,^^5^	0.31 (0.02)^1^^,^^5^	0.31 (0.02)^1^^,^^5^	0.31 (0.01)^5^	0.38 (0.02)^1^^,^^2^^,^^3^^,^^4^	30.25	<.01
	FD (m)	2.90 (0.05)^2^^,^^3^^,^^4^^,^^5^	2.96 (.07)^1^^,^^3^^,^^4^^,^^5^	2.95 (.06)^1^^,^^2^^,^^4^^,^^5^	2.89 (.07) ^1^^,^^2^^,^^3^^,^^5^	2.86 (.06)^1^^,^^2^^,^^3^^,^^4^	5.60	<.01
Underwater	GT (s)	0.54 (.03)^2^^,^^3^	0.51 (0.02)^1^^,^^5^	0.51 (0.02)^1^^,^^5^	0.53 (0.03)	0.55 (0.01)^2^^,^^3^	5.25	<.01
	GD (m)	2.10 (0.10)^2^	2.04 (0.07)^1^^,^^4^^,^^5^	2.05 (0.07)^4^^,^^5^	2.11 (0.07)^2^^,^^3^	2.14 (0.06)^2^^,^^3^	5.60	<.01
	T5 (s)	1.55 (0.06)^2^^,^^3^^,^^5^	1.44 (0.06)^1^^,^^4^^,^^5^	1.44 (0.08)^1^^,^^4^^,^^5^	1.50 (0.10)^2^^,^^3^^,^^5^	1.70 (0.03)^1^^,^^2^^,^^3^^,^^4^	30.87	<.01

FKA (°), front knee angle; FAA (°), front ankle angle; FHA (°), front hip angle; RKA (°), rear knee angle; RAA (°), rear ankle angle; RHA (°), rear hip angle; BT (s), block time; TA (°), take-off angle; EA (°), entry angle; FT (s), flight time; FD (m), flight distance; GT (s), glide time; GD (m), glide distance; T5 (s), time to 5-m; SD, standard deviation; DKS, starting block double kick start front pedal angle/rear pedal angle; OSB12, starting platform; RM ANOVA, repeated measures ANOVA; *p* , statistical significance *p* < .05.

1 Significantly different to DKS 30°/30°, *p* < .05.

2 Significantly different to DKS 40°/40°, *p* < .05.

3 Significantly different to DKS 50°/50°, *p* < .05.

4 Significantly different to DKS 60°/60°, *p* < .05.

5 Significantly different to OSB12, *p* < .05.

### 3.1 Block phase

Post-hoc LSD tests revealed significant differences (*p* < .05) in lower-limb joint angles and block time between the DKS configurations and OSB12. The FKA angle was largest with OSB12 and decreased progressively as the DKS pedal angle increased (OSB12 vs. DKS60°/60°: MD = 38.91, *p* < .001, ES = 0.99; DKS30°/30° vs. DKS60°/60°: MD = 10.32, *p* < .001, ES = 0.90). The RKA angle showed the opposite pattern: it was highest in DKS30°/30° and lowest in OSB12 (DKS30°/30° vs. OSB12: MD = 41.15, *p* < .001, ES = 0.99), with significant differences across all configurations. The FAA angle increased with greater pedal angles, being smaller in DKS30°/30° than in DKS60°/60° (MD = –6.14, *p* < .001, ES = 0.70). RAA also increased with pedal angle on the DKS block, whereas OSB12 produced the lowest value; the largest difference was observed between DKS60°/60° and OSB12 (MD = 17.50, *p* < .001, ES = 0.94). For the hip angles, higher FHA and RHA values were observed at lower pedal angles (DKS30°/30°), whereas the lowest values were recorded with OSB12 (DKS30°/30° vs. OSB12: FHA MD = 12.01, *p* < .001, ES = 0.91; RHA MD = 28.98, *p* < .001, ES = 0.98). Block time (BT) was longest with OSB12 and shortest with DKS40°/40° and DKS50°/50°, with marked differences between OSB12 and these configurations (DKS40°/40° vs. OSB12: MD = –0.16, *p* < .001, ES = 0.90; DKS50°/50° vs. OSB12: MD = –0.16, *p* < .001, ES = 0.78). Additional significant pairwise differences are indicated in Table 1.

### 3.2 Flight phase

Both TA and EA decreased with increasing pedal angles on the DKS block, whereas OSB12 produced the highest values for these variables. The most pronounced differences in TA were observed between DKS60°/60° and OSB12 (DKS60°/60° vs. OSB12: MD = –3.96, *p* < .001, ES = 0.63), and a similar pattern was found for EA (DKS60°/60° vs. OSB12: MD = –4.52, *p* < .001, ES = 0.81). Flight time (FT) was longest in DKS30°/30° and OSB12 and shorter for the intermediate DKS configurations, with significant differences between DKS30°/30° and DKS40°/40° (MD = 0.02, *p* = .004, ES = 0.30) and between DKS50°/50° and OSB12 (DKS50°/50° vs. OSB12: MD = –0.07, *p* < .001, ES = 0.70). Flight distance (FD) showed slightly longer values for DKS40°/40° and DKS50°/50° compared with OSB12; the largest difference was found between DKS40°/40° and OSB12 (DKS40°/40° vs. OSB12: MD = 0.10, *p* < .001, ES = 0.42). Other significant pairwise differences are summarised in Table 1.

### 3.3 Underwater phase

For GT, no significant difference was detected between DKS30°/30° and OSB12 (DKS30°/30° vs. OSB12: MD = –0.01, *p* = .809, ES = 0.01), whereas DKS40°/40° showed a significantly shorter glide time than OSB12 (DKS40°/40° vs. OSB12: MD = –0.03, *p* = .002, ES = 0.45). GD followed a pattern similar to FD, with shorter distances at intermediate DKS configurations and the largest difference observed between DKS40°/40° and OSB12 (DKS40°/40° vs. OSB12: MD = –0.10, *p* < .001, ES = 0.42). The fastest 5-m times were obtained with the DKS40°/40° and DKS50°/50° configurations (both 1.44 s), whereas OSB12 produced the slowest T5 (1.70 s). Accordingly, the greatest differences in T5 were found between DKS40°/40° and OSB12 (DKS40°/40° vs. OSB12: MD = –0.26, *p* < .001, ES = 0.90) and between DKS50°/50° and OSB12 (DKS50°/50° vs. OSB12: MD = –0.26, *p* < .001, ES = 0.80). Remaining significant pairwise comparisons for GT, GD and T5 can be found in Table 1.

## 4 Discussion

The aim of this study was to examine and understand the differences in angular and temporal characteristics of the individual phases of the 5-m start when performed from the new double kick start (DKS) block under different pedal configurations, in comparison with the OSB12 starting platform, and to interpret how these differences influence the biomechanics and efficiency of the start in junior competitive swimmers.

### 4.1 Block phase

The construction of the DKS starting block was inspired by findings from sprint start mechanics in athletics, where pedal spacing and inclination are systematically adjusted to optimise the balance between horizontal and vertical force production (35, 36). In this context, our results show that swimmers using the DKS block adopted moderately flexed front and rear knee angles, generally comparable to values reported as effective in track sprinting (6, 37, 38). From a biomechanical perspective, such knee positions place the centre of mass relatively close to the blocks and allow the hip, knee, and ankle extensors to generate a large horizontal impulse over a short period, while still providing sufficient vertical impulse to clear the pool edge.

Compared with athletics starting blocks, both the DKS and OSB12 platforms are inclined at approximately 10°, whereas track blocks are typically set at 0° relative to the running surface. This design difference, together with task-specific demands (purely horizontal sprinting vs. horizontal–vertical projection into water), likely explains why swimmers in our study displayed slightly greater knee angles than track athletes, yet remained within a similar functional range. We also observed clear differences in knee and hip angles between starts performed from the DKS block and those from the OSB12 platform. With increasing pedal angles on the DKS, hip flexion decreased at both the front and rear leg, indicating a more “open” position of the hips and a higher pelvis. This configuration resembles the set position in athletics and is favourable for rapidly transferring force from the lower limbs through the trunk to the upper body at take-off. In contrast, the OSB12 platform was associated with more flexed hip positions, suggesting a less mechanically advantageous posture for explosive horizontal force production (12, 39, 40).

Findings from the sprint start research support the notion that pedal settings influence both force production and temporal characteristics of the block phase. While some authors reported no substantial change in overall block performance with common adjustments of pedal angles (41), others demonstrated that reducing pedal angles can increase block velocity without necessarily shortening the block phase (37, 42). In our study, block time was shortest with the DKS 40°/40° and 50°/50° configurations and longest with the OSB12 platform, indicating that the sprint-inspired DKS arrangement allowed swimmers to generate the required impulse more rapidly than the traditional swimming block. The absolute block times in our junior swimmers were still somewhat longer than those reported for elite track athletes, which can be explained by differences in push-off direction (horizontal vs. combined horizontal–vertical) and performance level (elite athletes vs. junior competitive swimmers) (1, 6, 7, 10, 11). When compared with swimming-specific studies, our block times for the OSB12 platform are consistent with previously reported values in junior and senior swimmers (13, 15, 16, 18), whereas the shorter times achieved with the DKS 40°/40° and 50°/50° configurations suggest that adjustable pedals provide a biomechanical advantage in the block phase for this population.

### 4.2 Flight phase

Changes in block-phase mechanics were reflected in distinct differences in the flight phase. Starts performed from the DKS block were characterised by slightly smaller take-off (TA) and entry (EA) angles than those from the OSB12 platform. A somewhat lower take-off angle at a given horizontal velocity is expected to increase the centre of mass's horizontal displacement, whereas excessively steep angles tend to waste vertical impulse and shorten the effective trajectory. The DKS configurations, particularly at 40°/40° and 50°/50°, therefore appear to have provided a more favourable compromise between vertical clearance and horizontal projection.

Across the different DKS settings, TA and EA values showed a slight decrease with increasing pedal angle. This pattern suggests that higher pedal angles encouraged swimmers to adopt a posture that projects the body into a shallower trajectory. When compared with previous research, the TA and EA values obtained with the DKS30°/30° configuration were very close to those reported for elite junior swimmers starting from OSB14 blocks (18), and they diverged progressively as pedal angles increased. These similarities indicate that the lower-range DKS configurations (e.g., 30°/30°) can reproduce technical parameters observed in elite juniors, whereas higher angles may be reserved for swimmers who can exploit a shallower entry while maintaining sufficient horizontal speed. In contrast, the markedly lower TA values reported for the fastest swimmers in the study by Wadrzyk et al. (39) suggest that at very high performance levels, even smaller take-off angles might confer an advantage, provided that the swimmer can maintain control and minimise hydrodynamic resistance upon entry.

The temporal and spatial characteristics of the flight phase further support the functional benefits of the DKS. In our study, the shortest flight times and the greatest flight distances were generally observed with mid-range DKS configurations (40°/40° and 50°/50°), whereas the OSB12 platform yielded longer flight times and shorter distances. This combination of shorter time and longer distance indicates that swimmers entered the water with a higher average horizontal velocity when using the DKS block, consistent with the more explosive block-phase mechanics described above. Comparisons with previous studies using OSB11, OSB14, or alternative blocks such as SO2-X (16, 18, 28, 39) suggest that the type and configuration of the starting block can meaningfully affect flight-phase performance, and that variable DKS settings allow coaches to approximate or even exceed flight performance reported for elite swimmers in some conditions.

### 4.3 Underwater phase

The underwater phase integrates the outcomes of both the block and flight phases and is crucial for determining overall start effectiveness. In our junior competitive swimmers, the shortest glide times were observed with the DKS40°/40° and DKS50°/50° configurations, while the OSB12 platform produced slightly longer glide times without a clear advantage in glide distance. This pattern suggests that swimmers entered the water with greater initial velocity when using the DKS block and were able to preserve this velocity over the first metres of the underwater trajectory. The greatest glide distances were recorded with OSB12 and the DKS60°/60° configuration, but these values need to be interpreted in the context of glide time and the passive nature of the underwater phase in our protocol.

When comparing our results with previous studies, methodological differences must be taken into account. We instructed swimmers to glide passively to 5 m without performing dolphin kicks, which isolates the effect of entry speed and body position but naturally limits glide distance. In contrast, studies such as those by Wadrzyk et al. (39) and Tor et al. (29) examined active underwater movement over longer distances (e.g., 15 m) and reported distances at which the first kick occurred, typically beyond 4 m. The shorter distances and different temporal patterns observed in our data are therefore not necessarily indicative of lower efficiency, but rather reflect a design focused on the initial passive component of the underwater phase. Within this context, the DKS configurations—especially 40°/40° and 50°/50°—appear to promote a more favourable balance between entry speed and hydrodynamic resistance than the OSB12 platform.

The integration of block, flight, and underwater phases is reflected in the 5-m start time (T5). In our junior competitive swimmers, the shortest T5 values were obtained with the DKS40°/40° and DKS50°/50° configurations, whereas the OSB12 platform produced clearly longer 5-m times. These findings confirm the influence of pedal angle and block design on start efficiency over 5 m and favour the DKS block. When compared with studies conducted over the same distance, our swimmers achieved T5 values equal to or shorter than those of junior and senior swimmers using standard OSB blocks (13, 15, 16). In studies where start performance was assessed over longer distances, such as 15 m, swimmers typically completed their first strokes before reaching 5 m (18, 19, 24), which complicates direct comparison. Nevertheless, taken together, the shorter block times, more favourable take-off and entry angles, and shorter 5-m times associated with the DKS40°/40° and DKS50°/50° configurations indicate that these settings may contribute to more efficient start performance in junior competitive swimmers.

From an applied perspective, our results suggest that the DKS block enables junior swimmers to adopt joint configurations that are biomechanically favourable for generating horizontal impulse, to achieve take-off and entry angles that support an efficient trajectory, and to translate these advantages into faster performance over the first 5 m of the race. The mid-range pedal angles (40°/40° and 50°/50°) appear particularly promising as a starting point for individual optimisation, although future research should examine how these configurations interact with strength, anthropometry, and technical level, and whether similar benefits are observed over longer distances and in different start types.

## 5 Conclusion

Based on the results of the kinematic analysis of the 5-m start, it can be concluded that the new DKS starting block with adjustable pedals—particularly the 40°/40° and 50°/50° configurations—enables junior performance swimmers to execute a more effective and faster start compared with the traditional OSB12 starting platform. The improvements stem primarily from the set position on the block, which resembles that of track and field sprint starts. Adjustments of the pedals on the DKS block led to shorter block time, enhanced flight phase performance (shorter FT and greater FD), and faster 5-m times. These findings suggest that the use of variable pedal angles on the new DKS starting block improves kinematic start parameters and positively affects 5-m start performance, which may have a beneficial impact on overall swimmer performance in the initial phase of a race.

### 5.1 Limitations

This study has several limitations. The research sample consisted only of male junior swimmers, which does not allow the findings to be generalized to female swimmers or other performance categories. Testing was conducted solely over a distance of 5 m, without evaluating longer segments in which the underwater phase and the first swimming strokes play a more prominent role. Another limitation is the absence of kinetic measurements, which would have provided a more detailed understanding of force distribution on the starting block. These limitations should be taken into account when interpreting the results, and future studies are recommended to include additional performance categories and more comprehensive measurements.

## Data Availability

The original contributions presented in the study are included in the article/Supplementary Material, further inquiries can be directed to the corresponding author.
